# Comparative Evaluation of Intraocular Lens Power Calculation Formulas in Children

**DOI:** 10.7759/cureus.24991

**Published:** 2022-05-14

**Authors:** Anju Rastogi, Kirti Jaisingh, Priyadarshini Suresh, Kamlesh Anand, Siddharth Baindur, Tanvi Gaonker

**Affiliations:** 1 Ophthalmology, Maulana Azad Medical College, Delhi, IND; 2 Ophthalmology, All India Institute of Medical Sciences, Jodhpur, Jodhpur, IND

**Keywords:** srk/t, hoffer q, holladay 1, barrett universal ii, pediatric cataract, intraocular lens power calculation

## Abstract

Introduction

With the advent of newer microsurgical techniques, the results for cataract surgery have become quite promising. An accurate intraocular lens (IOL) power calculation is one of the most important factors in optimizing the results. The aim of this study was to evaluate the accuracy of four IOL power calculation formulas, namely, Barrett Universal II, Holladay 1, SRK/T and Hoffer Q, using optical biometry in children undergoing cataract surgery with primary IOL implantation.

Methods

This was a cross-sectional study. A total of 60 eyes of 42 children aged 5-16 years with congenital cataract and having undergone uneventful cataract surgery with IOL implantation were included in the study. Eyes were divided into three groups based on the axial length (AL): short (AL <22.00 mm), medium (AL 22-24.50 mm) and medium long (AL 24.50-26.00 mm). Optical biometry was used and the IOL power was calculated using the Barrett Universal II formula. The predicted postoperative refraction with the other three formulas, namely, SRK/T, Holladay 1 and Hoffer Q, using the same IOL power was estimated. This was compared with the actual postoperative refraction (spherical equivalent at 12 weeks) to give the absolute prediction error. The mean of all absolute prediction errors gave the mean absolute prediction error (MAE) values for each formula that were then compared.

Results

The MAE was 0.64 ± 0.73 for Barrett Universal II, 0.7 ± 0.72 for Holladay 1, 0.71 ± 0.65 for Hoffer Q and 0.8 ± 0.75 for SRK/T. Thus, Barrett Universal II had the lowest MAE across the whole group. The difference in the MAEs was not statistically significant.

Conclusion

Barrett Universal II had the lowest MAE and thus was predictable for the highest number of eyes in our study, although this was not statistically significant (p=0.176).

## Introduction

Modern day cataract surgery has become more of a refractive procedure rather than merely a lens extraction procedure. With the advent of newer microsurgical techniques, the results have become quite promising. An accurate intraocular lens (IOL) power calculation is one of the most important factors in optimizing the results. The formulas for the same have evolved considerably through various generations. Earlier formulas were reliable only in eyes with average axial lengths (ALs). The newer formulas such as the Holladay 1, Hoffer Q, SRK/T (third-generation formulas) and Barrett Universal II, Haigis, Holladay 2 and the Olsen formulas (fourth-generation formulas) were designed to improve the refractive accuracy by increasing the number of parameters they assess.

Although these newer formulas have proved to be fairly accurate in adult eyes, their accuracy in pediatric eyes is still debatable [1]. Considering the fact that pediatric eyes are not just eyes with shorter ALs, but also with numerous anatomical differences and constantly changing parameters, the choice of formula becomes extremely difficult. Several authors have studied the accuracy of these formulas in children, but the results are inconsistent [2-5]. Also, most of the studies are retrospective. There is still no consensus as to which formula would give the least prediction error (PE) in such eyes and, there is also paucity of literature comparing fourth-generation formulas, such as the Barrett Universal II, with previous generation formulas in this age group.

Our study aimed to compare the mean absolute prediction errors (MAEs) of four IOL calculation formulas, namely, Barrett Universal II, Holladay 1, SRK/T and Hoffer Q, in a pediatric population undergoing cataract surgery with primary IOL implantation. The prediction errors were correlated with the AL.

## Materials and methods

A cross-sectional study was conducted in the pediatric ophthalmology department of a tertiary eye care centre (Maulana Azad Medical College, Delhi, India). Approval was taken from the Maulana Azad Medical College Institutional Ethics Committee (MAMC-ETH-04112017) and the research adhered to the tenets of the Declaration of Helsinki.

A total of 60 eyes of 42 children in the age group of 5-16 years with visually significant congenital or developmental cataract were enrolled in the study after taking informed consent from parents/local guardians. Patients with pre-existing glaucoma, uveitis, posterior segment pathology, corneal abnormalities, astigmatism of more than 3 diopters, traumatic cataracts were excluded from the study. Children uncooperative for office measurements were also excluded. A thorough pre-operative ophthalmic examination was performed including the estimation of best corrected visual acuity (BCVA) using Early Treatment Diabetic Retinopathy Study (ETDRS) chart and assessment of intraocular pressure (IOP) using noncontact tonometry (Shin-Nippon NCT-10; Rexxam, Osaka, Japan). A dilated fundus examination was carried out and an ultrasound B scan was performed whenever the fundus was not visible. IOL power calculation was done with Lenstar L5 900 (Haag-Streit, USA) and ultrasound A scan (Sonomed; Sonomed Inc., Lake Success, NY) using an average speed of 1550 ± 2 m/s, wherever Lenstar was not possible.

The parameters assessed were keratometry, axial length, anterior chamber depth (ACD), white-to-white diameter and lens thickness. Target refraction was determined according to the age of the patient and fellow eye status. To increase the accuracy of office measurements, an average of five readings were taken. Inter-observer errors were minimised by using a single experienced observer for all biometry and refractive error measurements. Children aged five to eight years of age were left with a hyperopic error to account for the myopic shift that occurs at ocular maturity.

The IOL power was calculated using the Barrett Universal II formula as the standard and the predicted postoperative refraction was noted. The IOL power thus obtained was used to back-calculate the predicted postoperative refraction with the Holladay 1, Hoffer Q and SRK/T formulas.

Patients were followed up for a period of 12 weeks. Slit-lamp examination, visual acuity assessment and fundus examination were done on the first postoperative day, and at weeks 1, 2, 4, 8 and 12. The assessment of refraction, IOP and BCVA was done at weeks 4, 8 and 12. At the end of 12 weeks, refraction was done by retinoscopy and refined by subjective refraction. It was then converted into spherical equivalent. The absolute difference between the actual postoperative refraction (in spherical equivalent) and formula-predicted postoperative refraction gave the absolute prediction error (PE) for that particular formula. This was irrespective of the sign of the prediction error. The mean of all the prediction errors gave the mean absolute prediction error (MAE) for each formula, which were then compared: Absolute prediction error = Formula-predicted postoperative refraction - Actual postoperative refraction. For the purpose of analysis, eyes were divided into three groups based on the axial length: short eyes, AL <22.00 mm; medium eyes, AL 22.01-24.50 mm; medium-long eyes, AL 24.51-26.00 mm. The formula with the least MAE overall and across all ALs was determined.

Surgical technique

All surgeries were performed by the same surgeon under complete aseptic precautions. Clear corneal incisions of 2.8 mm were made. Anterior continuous curvilinear capsulorrhexis (ACCC) of 5-mm diameter was done. This was followed by bimanual lens matter aspiration and in-the-bag IOL implantation of a single-piece acrylic foldable IOL. Main wound and side ports were sutured with 10-0 Vicryl. Intracameral moxifloxacin was injected and subconjuctival gentamycin and dexamethasone were given at the end of the surgery.

Data analysis

Quantitative variables were expressed as means; analysis of variance (ANOVA) test was done to have pairwise comparisons between the formulas. Logistic regression was used to correlate MAE with AL. A p-value <0.05 was considered statistically significant. SPSS Statistics, version 17.0 (SPSS Inc., Chicago, IL) was used for statistical analysis.

## Results

A total of 60 eyes of 42 children aged 5-16 years (mean 8.53 ± 3.43 years) with visually significant congenital or developmental cataract undergoing cataract surgery with primary IOL implantation were enrolled in the study. The various demographic variables are listed in Tables 1-2.

**Table 1:** Age distribution of patients

Age (years)	Number of patients	Percentage
5-6	22	36%
7-8	14	23%
9-10	8	13%
>10	16	26%

**Table 2:** Sex distribution of patients

	Frequency	Percentage
Female	28	46.67%
Male	32	53.33%
Total	60	100.00%

The preoperative BCVA ranged from 0.78 to 1.78 logarithm of minimum angle of resolution (logMAR) units with the mean being 1.41 ± 0.33 logMAR units. The mean postoperative BCVA at 12 weeks postoperatively was 0.52 ± 0.4 logMAR units. There was a statistically significant improvement in the postoperative BCVA (p<0.0001). The mean spherical equivalent was 0.52 ± 0.4 D. The axial length ranged from 20.64 to 25.69 mm with a mean of 22.75 ± 1.39 mm; 26.67% eyes had a short AL, 56.66% eyes had a medium AL and 16% eyes had a medium-long AL (Table 3). The keratometric power ranged from 39.12 to 47.62 D with a mean of 44.34 ± 1.82 D.

**Table 3:** Distribution of axial length

Axial length (mm)	Frequency	Percentage
<22.00	16	26.67%
22.01-24.50	34	56.66%
24.51-26.00	10	16%
Total	60	100%

The MAE was calculated for each formula as shown in Table 4. In this study, overall, the Barrett Universal II formula had the lowest MAE and SRK/T had the highest MAE. However, the difference in the MAEs among the different formulas was not statistically significant (p=0.176).

**Table 4:** Mean absolute prediction error for each formula

Formula	Mean absolute prediction error
Barrett Universal II	0.64 ± 0.73
Holladay 1	0.7 ± 0.72
SRK/T	0.8 ± 0.75
Hoffer Q	0.71 ± 0.65

In the short AL eye group, Barrett Universal II had the lowest MAE in 50% of the eyes. SRK/T had the lowest MAE in 25% of the eyes and Holladay 1 and Hoffer Q each had the lowest MAE in 12.50% of eyes. However, as the p-value was 0.392, it was not statistically significant.

In the medium length eye group, Barrett Universal II and Hoffer Q each had the lowest MAE in 41.18% of the total, Holladay 1 in 11.76 % of the total and SRK/T in 5.88% of the total eyes. However, as the p-value was 0.065, it was not statistically significant. In the medium-long AL eye group, Barrett Universal II had the lowest MAE in 80% of the total eyes and SRK/T had the lowest MAE in 20% of the total eyes. However, as the p-value was 0.18, it was not statistically significant (Table 5).

**Table 5:** Distribution of lowest MAEs for each formula in different AL groups AL, axial length; MAE, mean absolute prediction error

AL of eyes	Percentage of eyes having the lowest MAE with a particular formula			
	Barrett Universal II	Holladay 1	Hoffer Q	SRK/T
Short AL (<22.00 mm)	50%	12.50%	12.50%	25.00%
Medium AL (22.01-24.50 mm)	41.18%	11.76%	41.18%	5.88%
Medium-long AL (24.51-26.00 mm)	80%	0%	0%	20%

The distribution of percentages of eyes with the MAE within +/- 0.25 D, +/- 0.26 to 0.50 D, +/- 0.51 to 1 D and > +/- 1 D for each formula was also determined (Table 6). Barrett Universal II had the highest percentage of eyes with the prediction error within +/- 0.25 D.

**Table 6:** Distribution of percentages of eyes with the mean absolute prediction error within +/- 0.25 D, +/- 0.26 to 0.50 D, +/- 0.51 to 1 D and greater than +/- 1 D for each formula

Prediction error	Barrett Universal II	SRK/T	Holladay 1	Hoffer Q
Within +/- 0.25 D	40%	20%	30%	20%
+/- 0.26 to 0.50 D	16.67%	26.67%	20%	26.67%
+/- 0.51 to 1 D	23.33%	26.67%	30%	36.67%
Greater than +/- 1 D	20%	26.67%	20%	16.67%

The mean PE was defined as the difference between the mean predicted postoperative refraction of each formula and the mean spherical equivalent. As is seen in Table 7, all the formulas resulted in a slight myopic error that was the lowest with the Barrett Universal II formula and maximum with the SRK/T formula, but as the p-value was >0.05, it was not statistically significant for any of the formulas.

**Table 7:** Mean prediction error for each formula

Formula	Mean prediction error	p
Barrett Universal II	-0.2125	0.2297
Holladay 1	-0.2515	0.1692
Hoffer Q	-0.2295	0.1923
SRK/T	-0.3208	0.1079

## Discussion

Paediatric eyes differ from adult eyes in various respects. They undergo axial elongation, corneal flattening and reduction in lens thickness as they grow [1]. Thus, determining an accurate IOL power in children is very challenging. Lack of fixation and correct centration during calculations add to the challenge. Various formulae are in vogue for IOL power calculation in children. However, there is still confusion as to which is the best formula that would give the lowest postoperative prediction error.

The predictive accuracy of newer generation formulae like Barret Universal II has been proven in adults by several studies [6-9]. But to the best of our knowledge, no study has been conducted to test its accuracy in this age group.

There was a statistically significant improvement in the BCVA postoperatively. The mean preoperative BCVA was 1.41 ± 0.33 logMAR units. The mean postoperative BCVA at four weeks was 0.54 ± 0.4 logMAR units (p<0.0001) and at 12 weeks was 0.52 ± 0.4 logMAR units (p<0.0001). The refractive error and BCVA stabilised by 12 weeks at which time the spherical equivalent was calculated.

In a study by Kane et al., in adult eyes, seven IOL power formulas were compared (Barrett Universal II, SRK/T, T2, Hoffer Q, Holladay 1, Holladay 2, Haigis) using optical biometry techniques [7]. Our observations mirror their findings in that the Barrett Universal II formula had the lowest MAE over the entire axial length range. In his study, Barrett Universal II was the most accurate formula for medium, medium-long and long eyes. There was no statistically significant difference in the MAE in the short AL group. In another study by Cooke and Cooke, nine IOL power formulas were compared using optical biometry in adult eyes only [8]. These formulas included Barrett Universal II, SRK/T, Haigis, Holladay 1, Olsen and Superformula. The Olsen formula was the best with Lenstar, while Barrett Universal II performed the best with IOL Master. Roberts et al. compared the latest artificial intelligence formula, the Hill Radial Basis Function (Hill-RBF) with the Barrett Universal II and existing third- and fourth-generation formulas [9]. The Hill-RBF and Barrett Universal II formulas provided the lowest MAE compared with existing formulas in short and long eyes, respectively. The Barrett Universal II formula had the lowest percentage of refractive surprises (>1.0 D from the predicted error) across all axial lengths. Similarly, in this study, Barrett Universal II had the maximum percentage of eyes with MAE within +/- 0.25 D.

Vasavada et al. did a similar study in paediatric eyes using Holladay 2, Holladay 1, Hoffer Q and SRK/T, and found that SRK/T and the Holladay 2 formulae had the lowest PE especially in extremely short eyes [5]. Nihalani and VanderVeen showed Hoffer Q to be the most predictable among SRK II, SRK/T and Holladay 1 in children [2]. However, they didn’t compare Barrett Universal II in their cohort. In this study, the Holladay 1 was the next best performing formula after the Barrett Universal II formula. It did not show a statistically significant difference over the other formulas.

Our results for the third-generation formulas (Holladay 1, SRK/T and Hoffer Q) agree with the results of the large study by Aristodemou et al. in which the Holladay 1 formula resulted in the lowest MAE in eyes with a medium axial length [10]. Hoffer also found that the Holladay 1 formula had the lowest MAE for medium-length eyes compared with the Hoffer Q, SRK/T and Holladay 2 formulas [11].

In this study, the mean absolute prediction error for Barrett Universal II was 0.64 ± 0.73, for Holladay 1 was 0.7 ± 0.72, for SRK/T was 0.8 ± 0.75 and for Hoffer Q was 0.71 ± 0.65. Thus, Barrett Universal II had the lowest MAE across the whole group. However, the p-value was 0.176 (>0.05), which is not statistically significant.

Hoffer Q is the most popular formula for use in eyes with an AL of 22.0 mm or less [11]. It has been reported as the most accurate formula for short eyes in studies by Hoffer (although the study included only 10 eyes with a short AL), Gavin and Hammond (although it was tested only against the SRK/T), and Aristodemou et al. (although only in eyes with an AL <21.0 mm) [10-12]. In contrast, Olsen and Hoffmann noted a tendency for the SRK/T and Holladay 1 formulas to have lower MAEs than the Hoffer Q and Haigis formulas [13]. In our study, Barrett Universal II performed the best in eyes with AL <22 mm (short eyes) that comprised 26.66% of the total eyes (16 eyes of 60) and Hoffer Q and Holladay 1 performed the worst.

In our study, 56.66% of eyes (34 eyes of 60 eyes) had an axial length within 22-24.5 mm (medium length). The Barrett Universal II and Hoffer Q formulas fared the best and SRK/T the worst in this range. However, as the p-value was 0.065, it was not statistically significant. In the study by Kane et al., Barrett Universal II was the most accurate formula for medium-length eyes [7]. In Barrett’s original publication, this formula was considered more accurate than the other third-generation formulas [6].

The most accurate IOL power formula in medium-long (24.5-26 mm) eyes was previously not clearly defined. Hoffer found the SRK/T formula to be the most accurate (followed by Holladay 1) [11]. Aristodemou et al. showed the Holladay 1 formula to be more accurate than both the SRK/T and Hoffer Q formulas [10]. Narváez et al. found no difference between the formulas [14]. Wang and Chang found that the Haigis formula was the most accurate [15]. In our study, 16% of eyes had an axial length between 24.5 and 26 mm. Among the 10 eyes, the MAE of Barrett Universal II was the lowest in eight eyes (80%) and the MAE of SRK/T was the lowest in two eyes (20%). However, as the p-value was 0.18, it was not statistically significant.

One of the major limitations of our study is the small sample size. Due to time constraint, only 60 eyes could be evaluated. Hence, only a small number of patients from each axial length group could be studied and the results are therefore not statistically significant. Children below five years were not included in the study to remove any confounding factors of posterior capsulotomy on surgical technique.

## Conclusions

Although the difference in the MAEs of the formulas was not statistically significant, the Barrett Universal II formula was found to be predictable for the highest number of eyes and gave the lowest MAE in short, medium and medium-long eyes as compared to SRK/T, Hoffer Q and Holladay 1. To our knowledge, this is one of the first few studies evaluating a novel formula for pediatric IOL power calculation with successful results over a wider range of axial lengths. Future studies with a larger sample size are required to elucidate its exact repeatability in children. This would give a clue to the surgeons worldwide as to which formula to depend upon while calculating IOL power in children.
